# Network pharmacology combined with molecular docking to explore the potential mechanisms for the antioxidant activity of *Rheum tanguticum* seeds

**DOI:** 10.1186/s12906-022-03611-3

**Published:** 2022-05-03

**Authors:** Lingling Wang, Feng Xiong, Shuo Zhao, Yang Yang, Guoying Zhou

**Affiliations:** 1 Key Laboratory of Tibetan Medicine Research, Northwest Institute of Plateau Biology, Chinese Academy of Sciences, Xining, 810008 China; 2 University of Chinese Academy of Sciences, Beijing, 100049 China; 3 China Academy of Chinese Medical Sciences, Beijing, 100700 China

**Keywords:** *Rheum tanguticum*, Network pharmacology, Molecular docking, Antioxidant activity, LC-Q-TOF/MS

## Abstract

**Background:**

*Rheum tanguticum* (*R. tanguticum*) is an edible and medicinal plant that exhibits high antioxidant activity. The purpose of the present study was to investigate the bioactive components of its seeds and the potential mechanisms of antioxidant activity to provide a foundation for further developmental work on *R*. *tanguticum* seeds as a functional food.

**Methods:**

In this study, the antioxidant activities of *R. tanguticum* seeds were measured using DPPH, ABTS and FRAP assays. LC-Q-TOF/MS was used to identify the active compounds in the seeds, and Swiss Target Prediction was used to identify their potential targets. The DisGENET, DrugBank, OMIM and GeneCard databases were used to search for antioxidant-related targets.

**Results:**

The component–target–pathway network was constructed and included 5 compounds and 9 target genes. The hub genes included ESR1, APP, MAPK8, HSP90AA1, AKT1, MMP2, PTGS2, TGFB1 and JUN. The antioxidant activity signaling pathways of the compounds for the treatment of diseases were the cancer signaling pathway, estrogen signaling pathway, colorectal cancer signaling pathway, MAPK signaling pathway, etc. Molecular docking revealed that the compounds in *R*. *tanguticum* seeds could inhibit potential targets (AKT1, ESR1 and PTGS2).

**Conclusion:**

Molecular docking studies revealed that the binding energy score between liriodenine and PTGS2 was the highest (8.16), followed by that of chrysophanol (7.10). This result supports the potential for PTGS2-targeted drug screening and design.

**Supplementary Information:**

The online version contains supplementary material available at 10.1186/s12906-022-03611-3.

## Background

Antioxidation is the process of inhibiting the oxidation reaction caused by reactive oxygen species (ROS) [1]. ROS are reactive molecules derived from oxygen and include superoxide anions (O_2_^−^), hydrogen peroxide (H_2_O_2_) and hydroxyl radicals (OH^−^) [2]. ROS are produced mainly in the mitochondria as a byproduct of biochemical reactions [3]. Many chronic diseases, including cancer, Alzheimer’s disease and diabetes, are thought to be linked to ROS imbalance [4]. The effects of antioxidants are exerted via pathways of cell growth, apoptosis and differentiation and help to prevent and protect against diseases [5]. Research has increasingly suggested that antioxidants are biologically essential to combat ROS and that insufficient levels of antioxidants in the body can be complemented by food [6, 7]. Hence, research on antioxidants in food, especially those in plants, is of much interest [8, 9].

For thousands of years, traditional Chinese medicine (TCM) has been used to prevent and treat diseases by medications with multi-ingredient, multitarget and synergetic benefits [10]. A previous study suggested that components in *Fructus schisandrae* may act on different targets and have great potential in the treatment of various diseases by exerting antioxidant activity [1]. The potential molecular mechanisms of lychee seeds as anti-inflammatory and antioxidant agents have also been explored [11]. In a study of *Trigonella foenum-graecum* L. seeds, various compounds were found to be useful in reducing the lipid levels of blood. These compounds work on the insulin signaling pathway and have antioxidant potential [12]. Muzaffer Silinsin et al. studied the antioxidant capacity of *Inula graveolens* (L.) Desf by UHPLC–MS/MS and demonstrated that the significant antioxidant capacity of the plant might be related to the high abundance of phenolic compounds [13]. In a study of *Satureja boissieri*, the results showed that its flavonoid and phenolic contents were good natural sources for use in the food industry and pharmacological processes [14]. Therefore, TCM medications may increasingly be used as antioxidants as the modern pharmacological understanding of their mechanisms progresses.

*Rheum tanguticum* Maxim. ex Balf. (*R*. *tanguticum*), a perennial plant of the genus *Rheum* L. from the Polygonaceae family, is a well-known Chinese herbal medicine [15]. The roots have been used as folk medicine for thousands of years in many countries, especially China, Korea and Japan [16]. The stems have high medicinal value and are low in calories. They are popular worldwide as a food to aid weight loss. It is recorded in the *Medical Canon in Four Sections* (*Si Bu Yi Dian*) that the stem, when the stem bark had been peeled off, was traditionally eaten in the Tibetan diet. The seeds of *R. tanguticum* are regarded as a byproduct, and only small amounts of the seeds are used for breeding, while the rest are discarded as waste. Phytochemical research has shown that rhubarb seeds contain 17 amino acids, with a total amino acid content as high as 18.43% [17]. The seeds are also rich in polysaccharides and proteins [18]. Polysaccharides from plants are characterized as natural antioxidants with excellent antioxidant activity [19]. Emodin and rhein, derived from rhubarb seeds, have been reported to possess a wide range of antioxidant, antibacterial, anti-inflammatory and antimicrobial biological activities [20, 21]. Compared with synthetic antioxidants, natural antioxidants from medicinal plants are more desirable in terms of health. Therefore, seeds can be recommended as a novel natural source of antioxidants [22]. Given the antioxidant potential of *R. tanguticum* seed extract, it is important to evaluate its antioxidant activity, and further research on its chemical constituents is warranted. Several methods, including assays based on 2,2-diphenyl-1-picrylhydrazyl (DPPH), ferric ion reducing antioxidant power (FRAP) and 2,2′-azino-bis(3-ethylbenzothiazoline-6-sulfonic acid (ABTS), are commonly used for the determination of antioxidant activity [23]. For example, the antioxidant properties of *Inula discoidea* were determined by the ABTS, DPPH, and FRAP methods [24]. To the best of our knowledge, there are no previous studies on the antioxidant activities of *R. tanguticum* seed extracts.

The complexity of TCM ingredients makes it difficult to disentangle the molecular mechanisms of action on diseases. As a cross-discipline combining bioinformatics, computer simulation and molecular network data, network pharmacology is an emerging tool for TCM data mining [25, 26]. A component–target–pathway–disease network can be established to systematically explore the underlying mechanisms of TCM in treating disease. Molecular docking is widely used in computer-assisted drug design technology to estimate the major binding modes of small molecules with the 3D structure of the target protein [27]. Both techniques have been successfully applied in TCM to identify compounds with potential efficacy in treating diseases [28, 29]. Attempts toward this direction have already been reported for *R. tanguticum*, focusing on multiple active ingredients aimed at multiple targets [30]. However, systematically understanding how multiple therapeutic targets work together to exert antioxidant effects still needs further investigation.

Our study aimed to verify whether *R. tanguticum* seeds have antioxidant activity and to explain how the major components related to the antioxidant mechanisms work. Here, we measured the antioxidant capacity (DPPH, ABTS and FRAP) of *R. tanguticum* seeds and identified the bioactive metabolites in the seed extract through liquid chromatography–quadrupole time-of-flight mass spectrometry (LC-Q-TOF/MS). Furthermore, the component–target–pathway network was investigated using network pharmacology analysis to explore the potential molecular mechanisms of the antioxidants. Molecular docking studies for the identified compounds were then conducted to evaluate their therapeutic potential and facilitate the design of candidates for treating oxidative diseases.

## Materials and methods

### Plant materials and sample preparation

The seeds were obtained from *R. tanguticum* plants at the Haidong Agricultural Experiment Station (Qinghai, China). Authentication was provided by Prof. Guoying Zhou of the Northwest Plateau Institute of Biology. The seeds were ground into powder using a grinder, packed into sealed polyethylene bags and kept at 4 °C for further use.

The powder was precisely weighed to 1 g, and 20 mL distilled water was added to the tubes. Following sonication (30 °C, 90 min, 350 w), the samples were centrifuged at 4000 r/min for 10 min, and the supernatant was collected. The resulting residue was added to 5 mL distilled water and centrifuged again under the same conditions. The supernatants were combined, and the volume was made to 50 mL with distilled water. The resultant reagent was used for subsequent experiments.

### Chemicals and reagents

Total antioxidant capacity assay kits for ABTS and FRAP were acquired from the Nanjing Jian Cheng Bioengineering Institute (Nanjing, China). All other chemicals, including phosphoric acid and ethanol, were of analytical grade and provided by Chengdu Cologne Chemical Co., Ltd. (Chengdu, China).

### Determination of antioxidant capacity

#### DPPH free radical scavenging activity assay

The free radical scavenging capacity of *R. tanguticum* seeds was determined using DPPH following a modified method [31]. Sample solution (2 mL) was added to 0.2 mmol/L DPPH in ethanol solution (2 mL) and mixed. After reacting in the dark for 30 min, the absorption values at 517 nm were measured using a UV spectrophotometer (756PC, Shanghai, China). The DPPH radical scavenging ability was calculated according to Eq. (1):

1 $$\mathrm{DPPH}\ \mathrm{scavenging}\ \mathrm{activity}\%=\left(\ 1-\frac{\mathrm{A}\mathrm{i}-\mathrm{Aj}}{\mathrm{A}0}\ \right)\times 100$$

where *A*_*i*_ is the absorbance of the sample and *A*_0_ is the absorbance of the blank, in which the samples containing *R. tanguticum* seed extracts were replaced by anhydrous ethanol. Moreover, in the control group, DPPH was replaced by 2 mL anhydrous ethanol (*A*_*j*_). Vitamin C (VC) served as a positive control. Each experiment was performed in triplicate.

#### ABTS scavenging activity assay

Blue ABTS^+^ is inhibited by antioxidants, and the total antioxidant capacity of a sample can be calculated by reading the absorbance [32]. For the ABTS^+^ radical scavenging assay, the amount of ABTS was measured using an assay kit (A015–2-1). The kit included concentrated detection buffer, ABTS solution, substrate solution and Trolox solution. The ABTS working solution was made by mixing the first three reagents at a 76:5:4 ratio. Trolox is a water-soluble vitamin E analog and serves as an antioxidant standard [33]. Trolox (10 mM) was diluted to concentrations of 0.1, 0.2, 0.4, 0.8 and 1.0 mM with distilled water. The test sample solutions (10 μL) were added to 20 μL of substrate solution. Then, 170 μL of ABTS working solution was added to each well, and the absorbance was determined at 405 nm after reacting at room temperature for 6 min. Distilled water was used as a blank control. OD values were detected using an Epoch2 Microplate Spectrophotometer (BioTek, USA). Concentrations were plotted on the y-axis, while OD values were plotted on the x-axis. The ABTS concentration in each sample was calculated based on the standard curve (y = −0.928x + 1.0226, *R*^2^ = 0.9975).

#### Ferric ion reducing antioxidant power (FRAP) assay

The FRAP assay was carried out using a Total Antioxidant Capacity Assay Kit (A015–3-1) [34]. The FRAP working solution was prepared by mixing detection buffer, matrix fluid and substrate solution according to a 10:1:1 ratio. Meanwhile, 27.8 mg of FeSO_4_·7H_2_O was dissolved in 1 mL of distilled water. Subsequently, the standard solutions were serially diluted to concentrations of 0.15, 0.3, 0.6, 0.9, 1.2 and 1.5 mM. Sample solution (5 μL) was added to 180 μL of FRAP working solution in 96-well plates. The reaction mixtures were incubated for 3–5 min at 37 °C. The absorbance was then measured at 593 nm with an Epoch2 Microplate Spectrophotometer (BioTek, USA). Each sample was measured in triplicate, and the average value was calculated. A standard curve with the OD values on the y-axis and the concentration on the x-axis was plotted and yielded the following equation: y = 3.5851x − 0.6010, *R*^2^ = 0.9979. The absorbance of the sample was inserted into the equation to obtain the FRAP concentration.

### LC-Q-TOF/MS analysis

The seeds were analyzed qualitatively using LC-Q-TOF/MS. This part of the study was completed by Shanghai Weipu Chemical Technology Company in China.

### Network pharmacology analysis

#### Target collection and potential target prediction

The targets of the active compounds were predicted using Swiss Target Prediction (http://www.swisstargetprediction.ch/) [35].

GeneCards (https://www.genecards.org/) [36], DrugBank (https://www.drugbank.ca/) [37], DisGeNet (https://www.disgenet.org/) [38] and OMIM (http://www.omim.org/) [39] were used to select genes with ‘oxidative’ as the keyword. Venn diagrams (http://www.bioinformatics.com.cn/) were used to determine the intersection targets between compounds and disease.

#### Protein–protein interaction (PPI) and component–target–pathway network

Following intersection, targets were screened, and their PPI network diagram was established using STRING (https://string-db.org/) [40]). The component–target–pathway network was visualized using Cytoscape software (version 3.7.2).

#### GO enrichment and KEGG analysis

DAVID (https://david.abcc.ncifcrf.gov/) was used to perform GO function and KEGG (www.kegg.jp/kegg/kegg1.html) pathway enrichment analyses with *Homo sapiens* as the selected species [41–44]. This was visualized using the Weishengxin online tool (http://www.bioinformatics.com.cn/) [45]. The threshold level for all GO enrichment and pathway analyses was set as *p* < 0.05. The top-ranked pathways were integrated and plotted as the final pathway map.

### Molecular docking

The 2D structures of major active ingredients were retrieved from the *PubChem* (https://pubchem.ncbi.nlm.nih.gov/) database and saved as PDB files for further analysis [46]. The structure of the target protein (PDB format) was downloaded from the PDB database (https://www.rcsb.org/) [47]. Protein structures were hydrogenated, and water was removed using PyMOL (v2.1.0). The plugin PyVOL was used to explore and visualize binding pockets. Each ligand or small molecule was hydrogenated with AutoDock version 4.2.6. The size of the box was chosen to cover the potential active pocket. Molecular docking simulations were performed using Autogrid.

### Data analysis

Data are expressed as the mean ± SE. All of the extractions and analyses were performed in triplicate.

## Results

### Antioxidant capacity of *R. tanguticum* seed extracts

**Fig. 1:**
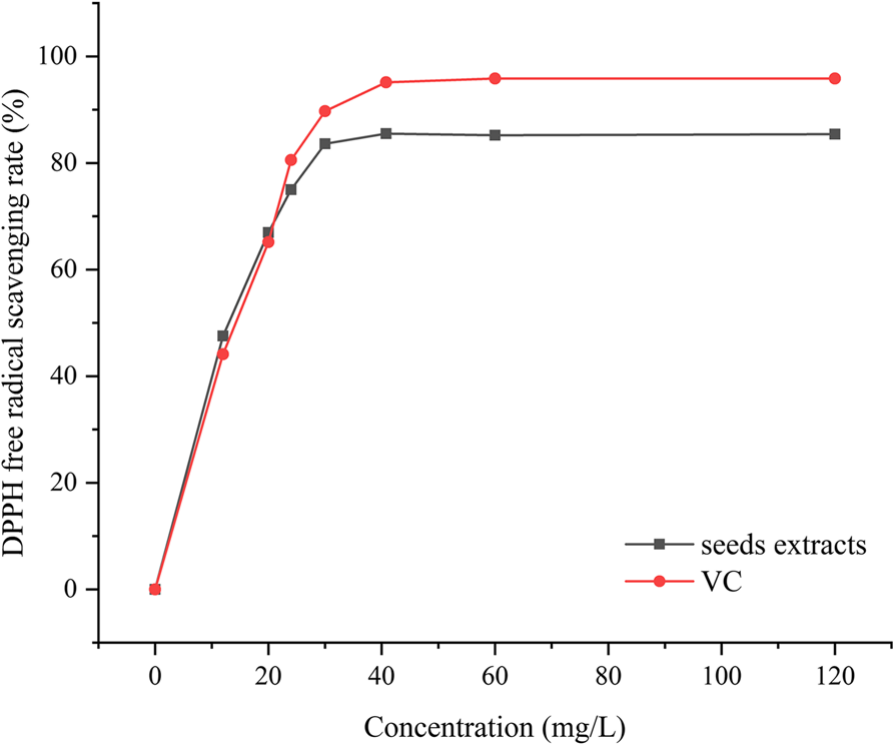
DPPH scavenging activity of *Rheum tanguticum* seed extracts

As shown in Fig. 1, as the seed extract and VC concentration increased, the DPPH free radical scavenging rate increased accordingly. When the concentration of VC in the positive control reached 60 mg/L, VC had the highest scavenging rate of the DPPH free radical, which was 95.8%, while the scavenging rate of *R. tanguticum* seed extracts plateaued at approximately 85%. However, the IC_50_ values were 12.31 and 15.06 mg/L, respectively. A higher rate of DPPH free radicals was obtained for *R. tanguticum* seed extracts in the concentration range of 12 ~ 20 mg/L compared to VC.

For the ABTS assay, Trolox was used as the standard, and the results were expressed as Trolox equivalents. For the FRAP assay, FeSO_4_·7H_2_O was used as the standard, and the results were expressed as FeSO_4_·7H_2_O equivalents. In our results, the ABTS radical cation scavenging ability and FRAP value of *R. tanguticum* seed extracts were 0.78 and 1.22 mmol/L, respectively (Table 1).

**Table 1:** Results of three antioxidant assays (DPPH radical scavenging activity, Vitamin C expressed in IC_50_; ABTS expressed in Trolox equivalent; FRAP expressed in FeSO4·7H2O equivalent)

DPPH (mg/L)	Vitamin C (mg/L)	ABTS (mmol/L)	FRAP (mmol/L)
12.31 ± 0.54	15.06 ± 0.24	0.78 ± 0.01	1.22 ± 0.11

### Identification of the metabolites in *R. tanguticum* seed extracts

The metabolites were extracted and analyzed using LC-Q-TOF/MS in both positive and negative ion modes. As shown in Table 2, 24 substances were identified, including kaempferol, propionic acid, emodin, chrysophanol and quercetin.

**Table 2:** Twenty-four compounds identified in *Rheum tanguticum* seeds

Metabolitecode	Identification	Formula	CAS
M1	Acetic acid	C_2_H_4_O_2_	64–19-7
M2	Propionic acid	C_3_H_6_O_2_	79–09-4
M3	Lactic acid	C_3_H_6_O_3_	50–21-5
M4	Pyruvic acid	C_3_H_4_O_3_	127–17-3
M5	Succinic acid	C_4_H_6_O_4_	110–15-6
M6	Malic acid	C_4_H_6_O_5_	6915-15-7
M7	Citric acid	C_6_H_8_O_7_	77–92-9
M8	Emodin	C_15_H_10_O_5_	518–82–1
M9	Chrysophanol	C_15_H_10_O_4_	481–74-3
M10	Emodin-3-methyl ether	C_16_H_12_O_5_	521–61-9
M11	D(+)-Glucose	C_6_H_12_O_6_	50–99-7
M12	Quercetin	C_15_H_10_O_7_	117–39-5
M13	Kaempferol	C_15_H_10_O_6_	520–18-3
M14	C.I. 75,710	C_21_H_20_O_12_	491–50-9
M15	Harpagoside	C_24_H_30_O_11_	19,210–12-9
M16	PINORESINOL DIGLUCOSIDE(P)	C_32_H_42_O_16_	63,902–38-5
M17	Betaine	C_5_H_11_NO_2_	107–43-7
M18	Rutin	C_27_H_30_O_16_.3(H_2_O)	153–18-4
M19	liriodenine	C_17_H_9_NO_3_	475–75-2
M20	THEAFLAVIN 3′-O-GALLATE	C_36_H_32_O_15_	28,543–07-9
M21	CATECHIN-(4ALPHA- > 8)-EPICATECHIN	C_30_H_26_O_12_	29,106–51-2
M22	L-Epicatechin	C_15_H_14_O_6_	490–46-0
M23	2,4,6-Trihydroxybenzoic acid	C_7_H_6_O_5_	83–30-7
M24	KUROMANIN CHLORIDE	C_21_H_21_ClO_11_	7084-24-4

### Network pharmacology analysis

#### Potential targets of the main active compounds and antioxidants

**Fig. 2:**
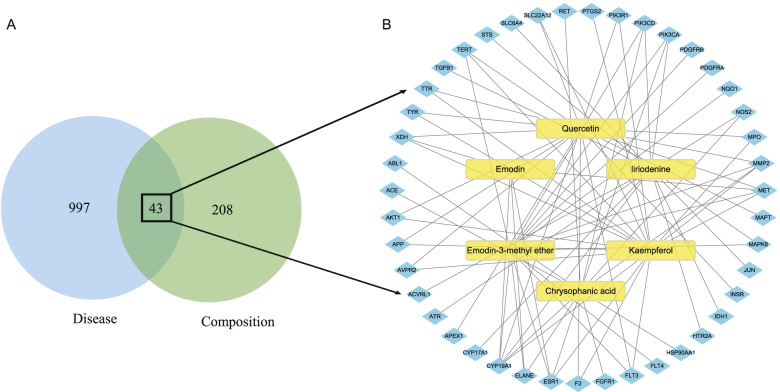
Venn diagram of active compound targets of *R. tanguticum* seeds and antioxidant targets (**A**) and interaction network between active components and intersection targets in *R. tanguticum* seeds (**B**)

Among the 24 ingredients in the seed extracts, 15 were selected by ADME (absorption, distribution, metabolism and excretion) and Lipinski’s rule of five screenings [48]. According to Swiss Target Prediction, 12 of the ingredients had a combined total of 261 potential targets with a probability larger than 0. Using ‘oxidative’ as a keyword, the related genes were selected from the disease gene databases (OMIM, GeneCards, DrugBank, DisGeNet). The results were pooled, and duplicates were deleted, leaving 1040 records to screen. Venn diagrams showed 43 intersections of oxidative-related and active ingredient targets (Fig. 2A). The intersection targets and corresponding compounds were imported into Cytoscape to draw a compound–target network diagram (Fig. 2B). This network contained 49 nodes linked via 80 edges, which revealed the synergistic multicomponent and multitargeted effects of *R. tanguticum* seeds contributing to their antioxidant activities.

#### PPI network of the targets

**Fig. 3:**
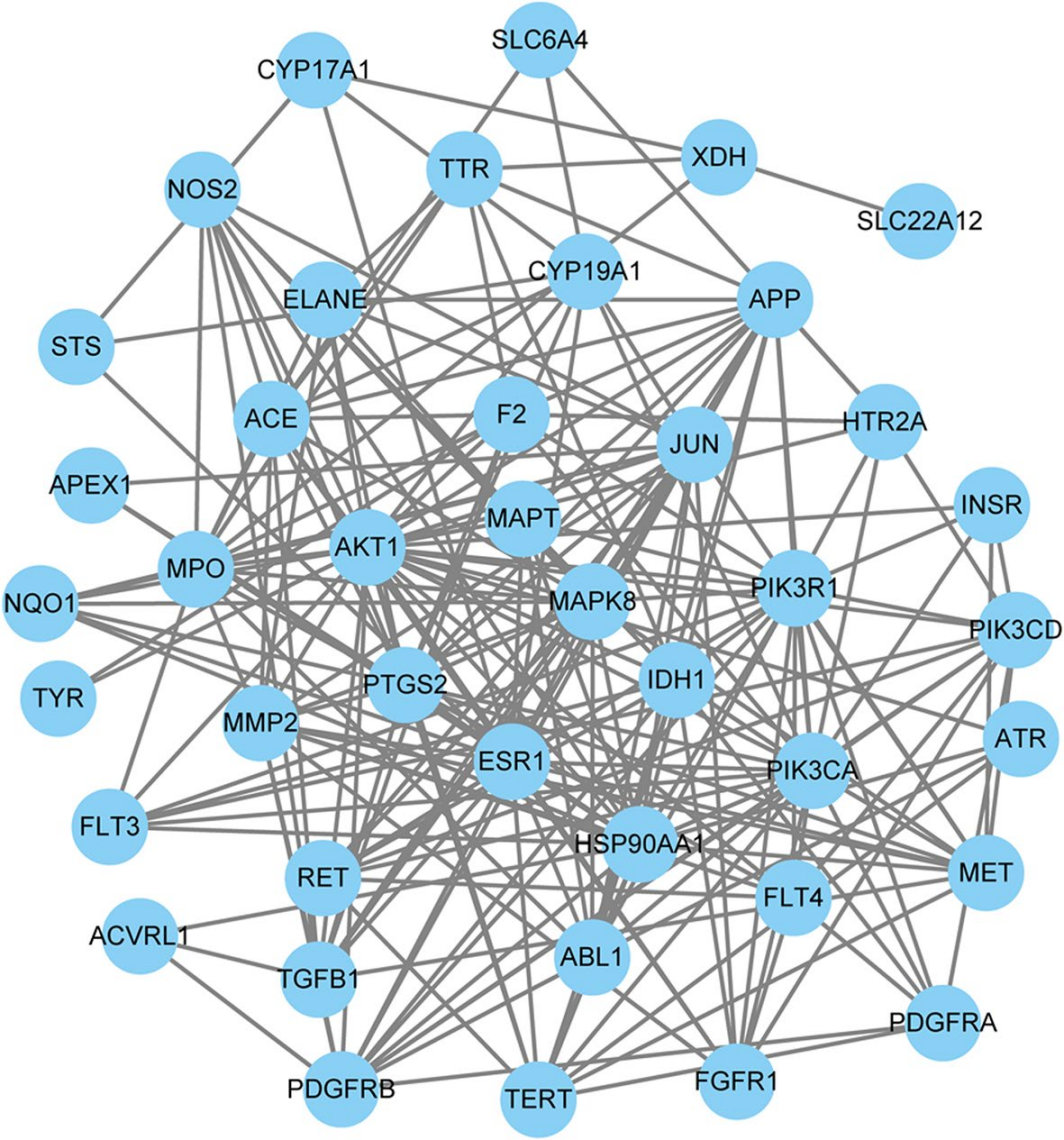
Protein–protein interaction (PPI) network of *R. tanguticum* seeds as a target for antioxidant treatment

**Fig. 4:**
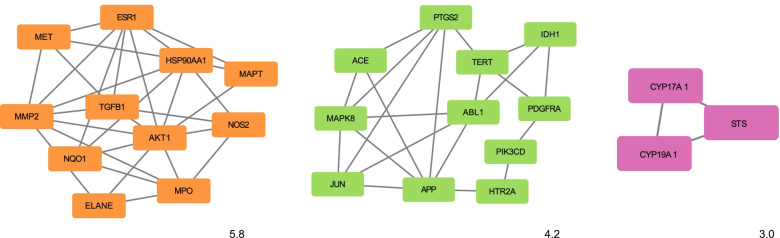
Modules in the PPI network of RTS for antioxidant treatment

To identify the hub genes in key modules, PPI network analysis was performed using the STRING database, and results with a combined score of ≥0.4 were selected (Fig. 3). The specific information is shown in Table 3. The top 10 hub genes were selected from the PPI network using the MCC algorithm and CytoHubba plugin. As shown in Fig. 4, the top three functional clusters of modules were selected (module 1, MCODE score = 5.8; module 2, MCODE score = 4.2; module 3, MCODE score = 3.0). Nine hub genes were identified by the intersection targets of MCC and MCODE (ESR1, APP, MAPK8, HSP90AA1, AKT1, MMP2, PTGS2, TGFB1 and JUN). The corresponding five metabolites were emodin, quercetin, liriodenine, chrysophanol and kaempferol. The results showed that a wide variety of substances could be involved in the antioxidant effects, such as receptors (vasopressin V2 receptor, estrogen receptor and others), proteins (microtubule-associated protein tau), hormones (transthyretin) and enzymes (steryl-sulfatase, telomerase reverse transcriptase, angiotensin-converting enzyme and others).

**Table 3:** Intersection target prediction between active components and disease

Number	Gene symbol	Target protein	Uniprot ID
1	ABL1	Tyrosine-protein kinase ABL1	P00519
2	ACE	Angiotensin-converting enzyme	P12821
3	ACVRL1	Serine/threonine-protein kinase receptor R3	P37023
4	AKT1	RAC-alpha serine/threonine-protein kinase	P31749
5	APEX1	NA-(apurinic or apyrimidinic site) endonuclease	P27695
6	APP	Amyloid-beta precursor protein	P05067
7	ATR	Serine/threonine-protein kinase ATR	Q13535
8	AVPR2	Vasopressin V2 receptor	P30518
9	CYP17A1	Steroid 17-alpha-hydroxylase/17,20 lyase	P05093
10	CYP19A1	Aromatase	P11511
11	ELANE	Neutrophil elastase	P08246
12	ESR1	Estrogen receptor	P03372
13	F2	Prothrombin	P00734
14	FGFR1	Fibroblast growth factor receptor 1	P11362
15	FLT3	Receptor-type tyrosine-protein kinase FLT3	P36888
16	FLT4	Vascular endothelial growth factor receptor 3	P35916
17	HSP90AA1	Heat shock protein HSP 90-alpha	P07900
18	HTR2A	5-hydroxytryptamine receptor 2A	P28223
19	IDH1	Isocitrate dehydrogenase [NADP] cytoplasmic	O75874
20	INSR	Insulin receptor	P06213
21	JUN	Transcription factor AP-1	P05412
22	MAPK8	Mitogen-activated protein kinase 8	P45983
23	MAPT	Microtubule-associated protein tau	P10636
24	MET	Hepatocyte growth factor receptor	P08581
25	MMP2	72 kDa type IV collagenase	P08253
26	MPO	Myeloperoxidase	P05164
27	NOS2	Nitric oxide synthase, inducible	P35228
28	NQO1	NAD(P)H dehydrogenase [quinone] 1	P15559
29	PDGFRA	Platelet-derived growth factor receptor alpha	P16234
30	PDGFRB	Platelet-derived growth factor receptor beta	P09619
31	PIK3CA	Phosphatidylinositol 4,5-bisphosphate 3-kinase catalytic subunit alpha isoform	P42336
32	PIK3CD	Phosphatidylinositol 4,5-bisphosphate 3-kinase catalytic subunit delta isoform	O00329
33	PIK3R1	Phosphatidylinositol 3-kinase regulatory subunit alpha	P27986
34	PTGS2	Prostaglandin G/H synthase 2	P35354
35	RET	Proto-oncogene tyrosine-protein kinase receptor Ret	P07949
36	SLC22A12	Solute carrier family 22 member 12	Q96S37
37	SLC6A4	Sodium-dependent serotonin transporter	P31645
38	STS	Steryl-sulfatase	P08842
39	TERT	Telomerase reverse transcriptase	O14746
40	TGFB1	Transforming growth factor beta-1 proprotein	P01137
41	TTR	Transthyretin	P02766
42	TYR	Tyrosinase	P14679
43	XDH	Xanthine dehydrogenase/oxidase	P47989

#### Gene Ontology (GO) and KEGG pathway analyses

**Fig. 5:**
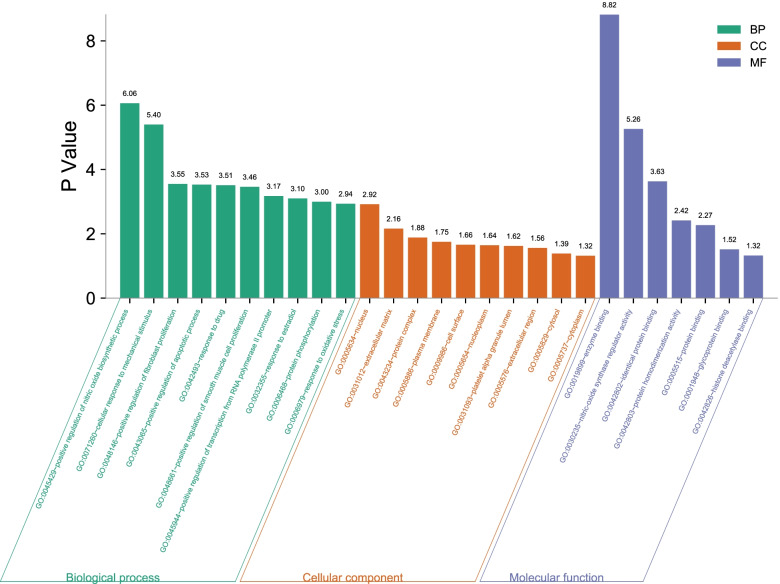
Biological process analysis of active components

**Fig. 6:**
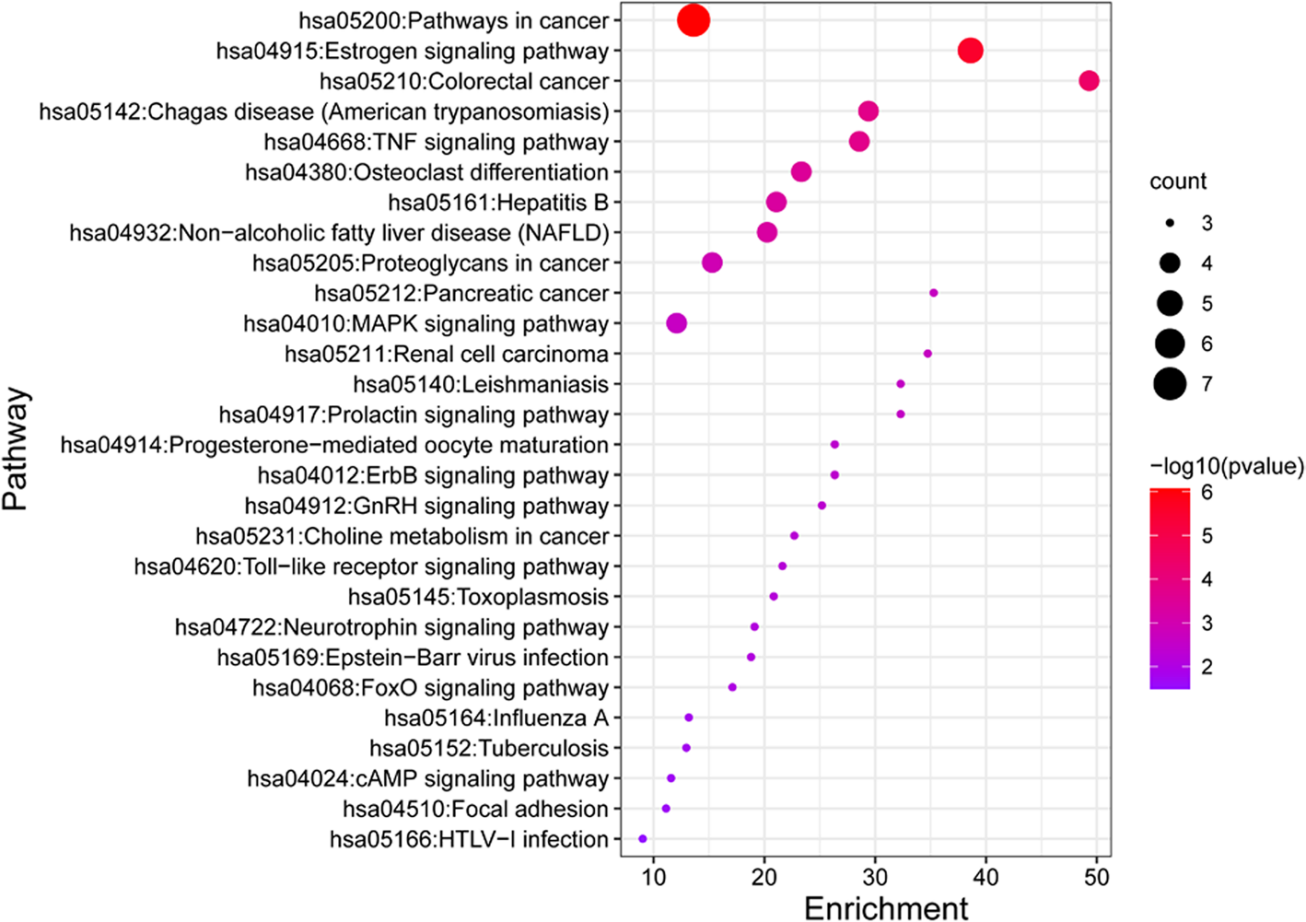
KEGG pathway enrichment analysis of active components

The DAVID 6.8 database was used for GO analysis and KEGG pathway enrichment of the nine key targets. Through GO analysis, a total of 70 GO items with *p* < 0.05 were obtained, including 53 biological process entries, 10 cell component entries and 7 molecular function entries. As shown in Fig. 5, the top 10 terms were arranged in ascending order of *p* values. The biological processes mainly included positive regulation of nitric oxide biosynthetic process, cellular response to mechanical stimulus and positive regulation of fibroblast proliferation. The cellular components that were enriched predominantly included the nucleus, extracellular matrix, protein complex and plasma membrane. The top molecular functions primarily included enzyme binding, nitric oxide synthase regulator activity, identical protein binding and protein homodimerization activity. Next, KEGG pathway enrichment analysis was conducted (Fig. 6). All of the pathways with a *p* value <0.05 were screened out and ranked by *p* value. The top three pathways were the cancer, estrogen signaling and colorectal cancer pathways.

#### Pathway–target–component network construction

**Fig. 7:**
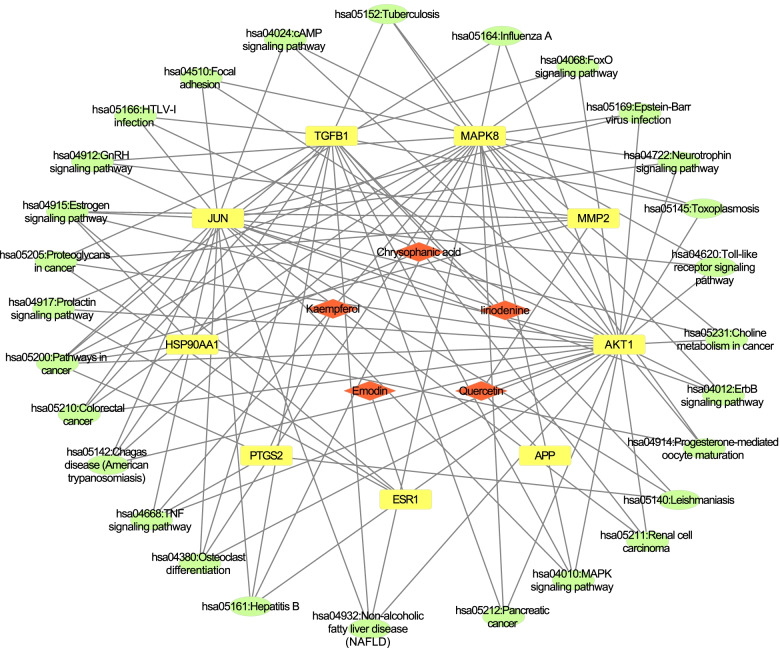
Active component-target-signal pathway network of *R. tanguticum* seeds

**Fig. 8:**
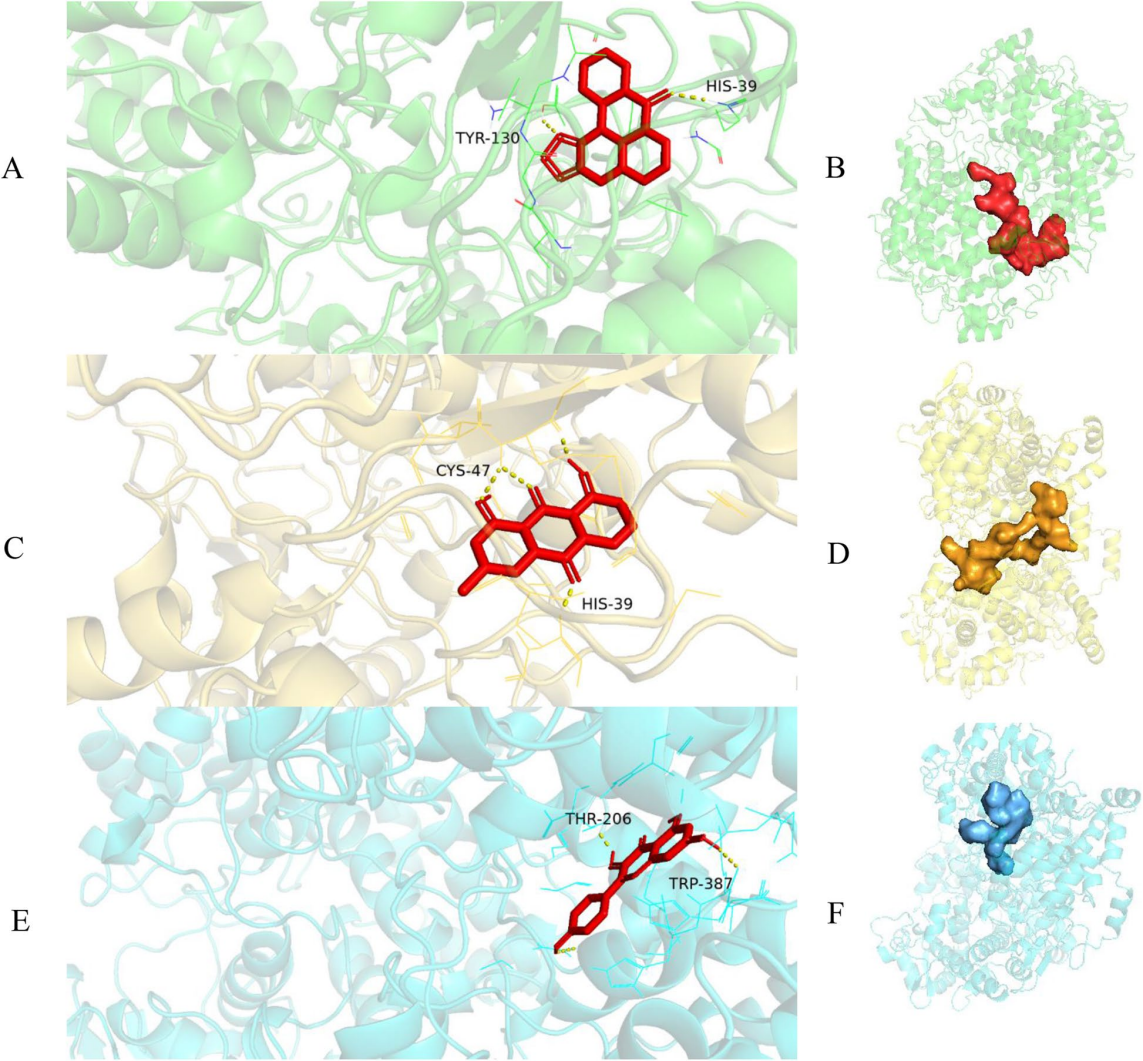
Molecular docking pattern and mapping surface showing molecules occupying the active pocket of proteins (**A** and **B**, PTGS2-liriodenine; **C** and **D**, PTGS2-chrysophanol; **E** and **F**, PTGS2-kaempferol)

The analysis of active ingredients, corresponding targets and signaling pathways is shown in detail in Fig. 7. From these results, emodin, quercetin, liriodenine, chrysophanol and kaempferol were the key components contributing to the antioxidant effect of *R. tanguticum* seeds. The top three key targets were ESR1, APP and MAPK8. Using the KEGG mapper tool to obtain the pathway map of *R. tanguticum* seeds in the treatment of oxidative disorders, we screened the top four pathways and integrated the final pathway map (Fig. 8). The target is marked in light color, while the potential target of the antioxidant is marked in dark color. The figure shows that the antioxidant targets of *R. tanguticum* seeds were scattered among the pathways of cancer, estrogen signaling, colorectal cancer and Chagas disease signaling and that the seed extract primarily functioned by regulating these pathways. Most targets could function in multiple pathways, such as JUN, AKT1 and MAPK8.

### Molecular docking of active components and key targets

**Fig. 9:**
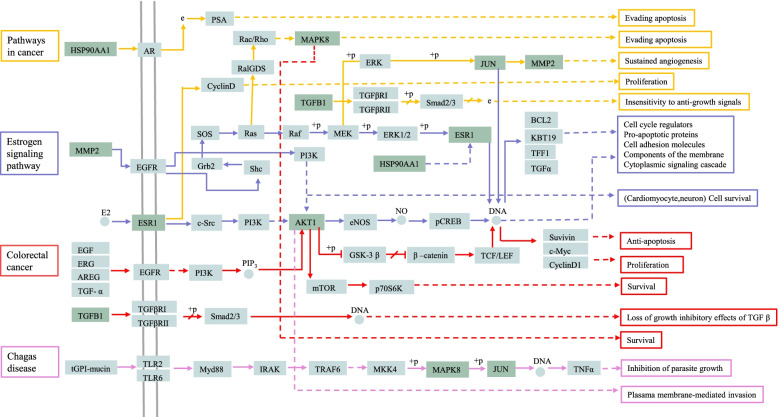
Potential antioxidant pathway of the main active ingredients of *R. tanguticum* seeds (↑ arrows indicate a promotion effect; T arrows indicate an inhibition effect). The target is marked in light color, and the potential antioxidant target of *R. tanguticum* seeds is marked in dark color

Five active ingredients and the top three targets ranked by degree were selected to conduct molecular docking (Table 4). Following convention, a binding capacity between the tested molecules and proteins was assumed to exist when the binding energy score was greater than 4.25. Scores greater than 5.0 indicate relatively high binding affinity, and scores greater than 7.0 indicate strong ligand–receptor interaction [49]. Hence, the selected active ingredients (liriodenine, chrysophanol and kaempferol) were docked one to one with PTGS2 using AutoDock according to the binding energy (>7.0). Liriodenine could form a hydrogen bond with PTGS2 at His-39 and Tyr-130. Chrysophanol could form a hydrogen bond with PTGS2 at Cys-47 and His-39, while kaempferol could form a hydrogen bond with PTGS2 at Thr-206 and Trp-387 (Fig. 9).

**Table 4:** Molecular docking results of active components from seeds and potential targets of antioxidant

Compound	Binding energy (kcal /mol)		
	AKT1	ESR1	PTGS2
Emodin	−5.16	−6.27	−6.65
Quercetin	−5.21	−5.65	−6.71
Liriodenine	−6.10	−6.49	−8.16
Chrysophanol	−5.69	−5.96	−7.10
Kaempferol	−5.04	−6.23	−7.08

## Discussion

Antioxidant activity has become a property of interest in functional foods. The use of natural antioxidants from plants (especially seeds), considered to be a substitute for synthetic antioxidants, has gradually become a trend [9]. Numerous studies have revealed that many components of *R. tanguticum* have significant antioxidant activities [40, 50]. Its seeds have a high soluble protein content and are rich in many amino acids and other nutritional components, according to previous data from our laboratory. Thus, it is of great significance to study the antioxidant mechanisms of *R. tanguticum* seeds.

The results of the three antioxidant assays verified that *R. tanguticum* seed extracts had strong scavenging effects on DPPH and ABTS radicals and high FRAP ability. The highest scavenging rate achieved by DPPH (85.5%) was lower than those previously reported for green tea and rosemary extracts (> 90%), which hold pioneering and instructive roles as natural antioxidants [51]. However, compared with the scavenging rate of the positive control (VC), the IC_50_ for *R. tanguticum* seed extracts was lower. This showed that within a specific concentration range, *R. tanguticum* seed extracts had better antioxidant activity than VC. Moreover, *R. tanguticum* seed extracts exhibited superior antioxidant activity (IC_50_ = 12.31 mg/L) when compared with goji, as the DPPH scavenging activity for the latter was 784 mg/L [52]. For ABTS radical scavenging, our results showed that the activity of *R. tanguticum* seed extracts (0.78 mmol/L) was lower than that of flaxseed meal (1.19 mmol/L) [53]. However, the total FRAP reducing ability was higher than those of grape seed oil (0.735 mmol/L) and *Origanum vulgare* subsp. *vulgare* essential oil (467.25 μmol/L) [54, 55]. The good antioxidant activity of *R. tanguticum* seeds may be attributable to the phenolics and flavonoids in the extracts. The LC-Q-TOF/MS qualitative results revealed that the main active components of our aqueous extract included flavonoids, polyphenols, alkaloids, anthocyanins and organic acids. Flavonoids and polyphenols are primary contributors to the antioxidative process [56, 57].

To determine the compounds primarily responsible for the antioxidant activity of *R. tanguticum* seeds, network pharmacology was then carried out. After Lipinski’s rule of five and the ADME filter process, 15 compounds were chosen for target prediction, and 5 of them were predicted to be core components. The PPI network was analyzed by MCODE to obtain the hub genes (ESR1, APP, MAPK8, HSP90AA1, AKT1, MMP2, PTGS2, TGFB1 and JUN). The ESR1 gene encodes an estrogen receptor alpha, and the antioxidant properties of estrogen might contribute to decreased oxidative stress [58, 59]. Chen et al. demonstrated that inflammation and oxidative stress phenomena occurred in the cerebrum and cortex of APP transgenic mice [60]. MAPK8/9/10 activation is an important upstream signal to trigger autophagy, and autophagy can rescue apoptosis caused by mild oxidative stress [11, 61]. The MAPK pathway lies upstream of JUN and can activate JUN expression. JUN is one of the components of AP-1, a transcription factor that regulates inflammatory response genes [62]. Growing evidence has revealed that flavonoids exert good anti-inflammatory effects as well as increased antioxidant activity [63]. Therefore, the antioxidant mechanisms of *R. tanguticum* seeds may be achieved by controlling inflammatory reactions and autophagy.

In the GO enrichment and KEGG pathway analyses, these genes were mainly enriched in the cancer, estrogen signaling and colorectal cancer pathways. Thus, it can be seen that the antioxidant effect is associated with multiple diseases. Relieving oxidative stress is an effective strategy to prevent or treat cancer and other diseases. *R. tanguticum* seeds may exert antioxidant effects through these signaling pathways and via biological processes such as the positive regulation of nitric oxide. In addition, the location of the target was assigned, and the pathway diagram was plotted, suggesting the pivotal role of hub genes (such as MAPK8, AKT1 and ESR1) in these pathways.

Molecular docking was used in this study to clarify the mechanism and provide valuable guidance for drug screening and design in future experiments. In our study, liriodenine was found to be one of the best choices for drug–target interactions with PTGS2. Liriodenine has been proven to have powerful antibacterial and antioxidant activities [64]. It shows strong activity in the prevention of dementia. However, the content of liriodenine in plants is very low (generally only 0.01%), which would be a bottleneck for the use of liriodenine in potential medicinal applications [65]. In our molecular docking experiment, chrysophanol also had a relatively high binding energy score with PTGS2 (7.10). According to previous studies, chrysophanol has antioxidant and senescence resistance properties and is being considered as a drug candidate for various neurodegenerative diseases [66, 67]. It is speculated that the hydroxyl (·OH) in chrysophanol conducts a one-electron conversion reaction with the superoxide anion (·O_2_^−^), which inhibits the autoxidation of pyrogallol and consequently exhibits a free radical scavenging effect. Therefore, chrysophanol may be a natural antioxidant that could be used as a food ingredient. More experiments in the future are required to verify this relationship and the mechanisms involved.

This study showed that *R. tanguticum* seed extracts produced antioxidant effects through synergism between multiple ingredients, targets and pathways. The mechanisms of action were elucidated through applied network pharmacology. These results suggest that the seeds may be used as a functional food supplement or further developed as a natural antioxidant.

## Conclusion

*R. tanguticum* seeds were shown to have good antioxidant capacity through DPPH, ABTS and FRAP assays. A total of 24 components in the seed extracts were identified by LC-Q-TOF/MS. The seed extracts were rich in flavonoids, organic acids, polyphenols and other substances. Five potential compounds and nine associated targets were identified using the network pharmacological approach. Emodin, quercetin, liriodenine, chrysophanol and kaempferol were the central components contributing to antioxidant activity. The core targets were ESR1, APP, MAPK8, HSP90AA1, AKT1, MMP2, PTGS2, TGFB1 and JUN. The crucial signaling pathways were identified to be cancer, colorectal cancer, estrogen signaling and MAPK signaling. In view of these results, the component–target–pathway network was constructed. A further integrated pathway diagram revealed that hub genes had core positions in the signaling pathway. These results might provide new insights for future research into the mechanisms behind the antioxidant effects of *R. tanguticum* seeds.

Molecular docking showed that all five potential drug compounds (emodin, quercetin, liriodenine, chrysophanol and kaempferol) had high binding energies to the predicted target proteins (AKT1, ESR1 and PTGS2). The binding energy score between liriodenine and PTGS2 was the highest (8.16), followed by that of chrysophanol (7.10). This result supports the potential for PTGS2-targeted drug screening and design.

## Supplementary Information

**Additional file 1.**

## Data Availability

All data generated or analysed during this study are included in this published article [and its supplementary information files].

## Abbreviations

*R. tanguticum*: *Rheum tanguticum*
TCM: Traditional Chinese Medicine
DPPH: 2,2-Diphenyl-1-picrylhydrazyl
ABTS: 2,2′-Azinobis-(3-ethylbenzthiazoline-6-sulphonate)
FRAP: Ferric ion reducing antioxidant power
PPI: Protein-protein interaction network
GO: Gene Ontology
KEGG: Kyoto Encyclopedia of Genes and Genomes
